# Two Cases of Dedifferentiated Endometrioid Carcinoma: Case Presentation and Brief Review of the Literature

**DOI:** 10.1155/2018/7624785

**Published:** 2018-09-25

**Authors:** Sachiko Morioka, Yasuhito Tanase, Ryuji Kawaguchi, Tomoko Uchiyama, Hiroshi Kobayash

**Affiliations:** ^1^Department of Obstetrics and Gynecology, Nara Medical University, Nara, Japan; ^2^Department of Diagnostic Pathology, Nara Medical University, Nara, Japan

## Abstract

Endometrioid carcinoma is the most common histological type of uterine endometrial cancer and particularly dedifferentiated endometrioid carcinomas (DEC) are less commonly observed. Silva et al. reported the biological features of UC based on the undifferentiated component of DEC, although the component represented only 20% of undifferentiated carcinoma. In this study, we report two cases of DEC with different presentation. Case 2 presented with the invasion to the bladder, rectum, and LN metastases. In contrast, the tumor in case 1 advanced into the endometrial cavity, similar to an endometrial polyp, without myometrial invasion. Hence, the diagnosis was established early. While we strive to improve the diagnosis of DEC, it is also crucial to better assess the prognosis and the appropriate treatment for the patients with established diagnosis of DEC.

## 1. Introduction

Endometrioid carcinoma is the most common histological type of uterine endometrial cancer, whereas serous, clear cell, undifferentiated, and particularly dedifferentiated endometrioid carcinomas (DEC) are less commonly observed. Silva et al. first reported the patients with undifferentiated uterine carcinoma (UC) coexisting with low-grade endometrioid carcinoma (grades 1 or 2) as having DEC in 2006 [1]. They reported the biological features of UC based on the undifferentiated component of DEC, although the component represented only 20% of UC. In this study, we report two cases diagnosed as DEC with different presentations. In the first case, the tumor developed on the surface of an endometrial polyp; in the second case, the tumor presented as an advanced cancer with metastasis in multiple lymph nodes.

## 2. Case Presentation

**Figure 1:**
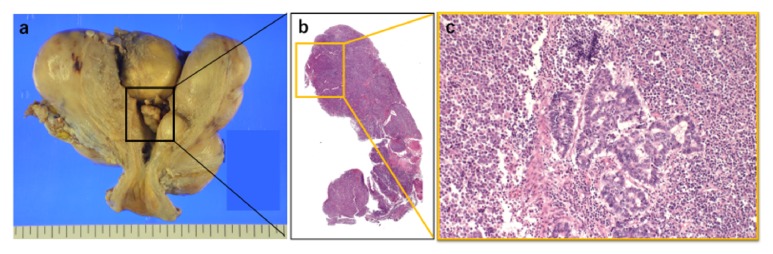
Case 1. (a) On gross examination a polypoid mass filled the endometrial cavity. (b, c) The tumor comprised well-differentiated endometrioid carcinoma and UC whose component represented about 80% of the whole neoplasm (hematoxylin and eosin [HE]).

**Figure 2:**
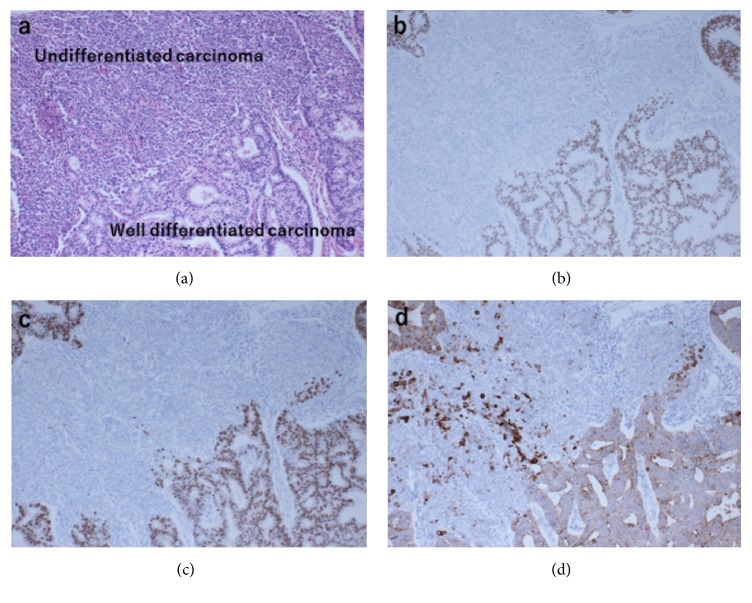
Case 1. (a) The tumor was composed of well-differentiated endometrioid carcinoma and UC with a sharp border (HE). ((b) ER, (c) PR) Differentiated components were strongly positive for ER and PR, whereas undifferentiated areas were not stained. (d) Pancytokeratin (AE1/AE3) was diffusely expressed in the well-differentiated carcinoma component and focally expressed in the UC component.

*Case 1.* A 68-year-old postmenopausal woman (gravida 2; body mass index [BMI], 32.4 kg/m^2^) presented at a local gynecology clinic 20 months ago with a chief complaint of vaginal spotting. Transvaginal ultrasonography showed no thickness of the endometrium, and endometrial cytology was negative. At the three-month follow-up visit, a repeat endometrial cytology was also negative. However, vaginal bleeding persisted, and the patient visited the clinic again a month ago. At this time, pelvic magnetic resonance imaging (MRI) was performed, which revealed irregularity and endometrial thickening, and the patient was referred to our institution—Nara Medical University, Kashihara, Nara, Japan—for further evaluation. Endometrial curettage was performed that revealed atypical cells with large nuclei and conspicuous nucleoli without gland formation, which appeared to be consistent with high-grade endometrioid carcinoma or UC. The level of tumor markers was not elevated: CA125, 17 U/ml; CA19-9, 9 U/ml; CA72-4, 2.9 U/ml; CEA, 1.1 ng/ml; and SCC, 0.9 ng/ml. Chest and abdominal contrast-enhanced computed tomography (CECT) revealed no metastatic lesions. Pelvic contrast-enhanced MRI showed multiple myomas and a 30 mm polyp-like mass projecting into the endometrial cavity without myometrial invasion. The patient underwent abdominal total hysterectomy, bilateral salpingo-oophorectomy, pelvic lymphadenectomy, para-aortic lymphadenectomy, and omentectomy. The surgical specimen of the uterus showed a 35 mm polypoid tumor developing from the uterine posterior wall. Microscopically, the polypoid tumor comprised well-differentiated endometrioid carcinoma, grades 1-2, and UC. The well-differentiated endometrioid carcinoma was confirmed on the surface of the endometrial polyp, and the coexisting UC showed a diffuse proliferation of atypical cells (Figure 1). Pancytokeratin (AE1/AE3) was diffusely expressed in the differentiated carcinoma component and was focally expressed in the UC component. Estrogen receptor (ER) and progesterone receptor (PR) were well expressed only in the differentiated carcinoma component (Figure 2). The UC component represented about 80% of the whole neoplasm. Endometrium invasion or lymph node (LN) metastasis was not observed. Based on these findings, the patient was diagnosed with DEC located on the endometrial polyp. The final Federation of Obstetrics and Gynecology (FIGO) stage was IA. The patient was treated with adjuvant chemotherapy (TC protocol: paclitaxel, 175 mg/m^2^ + carboplatin AUC 6, every three weeks, and six cycles). She has been disease-free for 15 months after the initial surgery.

**Figure 3:**
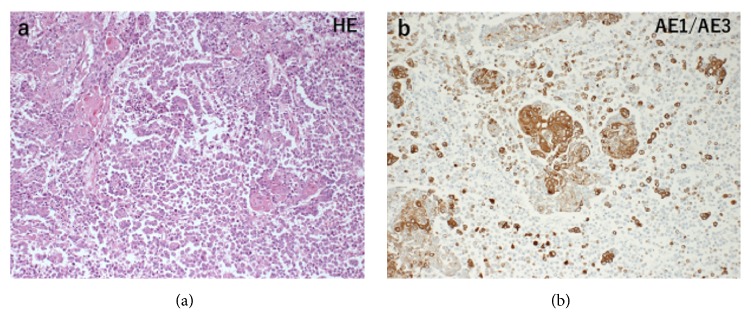
Case 2. (a) The tumor comprised moderately DEC and UC. (b) Pancytokeratin (AE1/AE3) was diffusely expressed in the well-differentiated carcinoma component and focally expressed in the UC component.

*Case 2.* A 58-year-old woman (BMI, 22.9 kg/m^2^), who had been hospitalized for several months with a diagnosis of bipolar disorder, reported that she has been experiencing atypical vaginal bleeding for >1 year, which had worsened over time. An abdominal CECT showed a large pelvic mass, and she was transferred to our institution for further evaluation. Pelvic MRI revealed a bulky mass in the whole uterine corpus, which spread to the bladder and rectum. Chest and abdominal CECT revealed multiple LN metastases, which extended from the para-aortic to pelvic LNs. Endometrial curettage revealed the foci of atypical cells arranged in sheets with numerous mitotic figures. There was no sarcoma component, and the histological pattern represented that of only a carcinoma. ER and PR tumor cell were focally expressed. As tumor markers, CA19-9, CEA, and SCC levels had risen (CA19-9, 43 U/ml; CEA, 13.9 ng/ml; SCC, 80.4 ng/ml); CA125 and CA72-4 levels were within normal range (CA125, 12 U/ml; CA72-4, 2.5 U/ml). Although the pathological diagnosis remained uncertain, based on the overall findings, the patient was diagnosed with stage IVA uterine endometrial cancer. Because of the presence of mental disorder and poor general condition (performance status 4), best supportive care was selected as the optimal treatment. However, the patient died in three months. Autopsy revealed uterine tumor invasion to the bladder, rectum, and pelvic wall with the involvement of the greater omentum and small intestine. The metastases to the pelvic and para-aortic LNs were observed. Microscopically, endometrioid carcinoma (grade 2) and UC components were present. Pancytokeratin (AE1/AE3) was diffusely expressed in the differentiated carcinoma component and focally expressed in the UC component (Figure 3). ER and PR tumor cells were expressed only in the differentiated carcinoma component. There were bone marrow hyperplasia and neutrophil infiltration in the lung and myocardium. The patient died of sepsis due to urinary tract infection secondary to the tumor invasion. The final diagnosis was DEC with FIGO stage IVB.

## 3. Discussion

DEC is defined as a tumor wherein the components of well-differentiated and UC are present. The transition between the two tumor components is abrupt with a sharp border. This histological pattern has also been reported in bone and soft tissue tumors such as chondrosarcoma [2], osteosarcoma [3], and liposarcoma [4]. In 2005, Silva et al. proposed classification criteria for endometrial uterine cancer [5]. In the following year, the authors presented the clinicopathological features of 25 cases of low-grade endometrioid carcinoma-associated UC and designated them as DEC [1]. They further examined the prognosis of these patients and concluded that DEC was associated with unfavorable prognosis, although the UC component was <20% of the whole neoplasm.

In 2014, the WHO classification of tumors of female reproductive organs (4th edition) added DEC as one of the pathological subtype of endometrial carcinoma [6]. Because the pathological diagnosis of DEC has only been established recently, the clinical features of the disease, including incidence, treatment, and prognosis, are unknown.

In the present study, we reviewed 68 cases associated with diagnosed DEC (Table 1) [1, 7–15]. Advanced cancer stages were higher among these patients than those observed in usual endometrial uterine cancers; 30 patients had stages I and II, and 38 patients had stages III and IV. Excluding the 30 cases that were not referred for the treatment, of the 38 remaining patients, hysterectomy was performed in 36 patients, leading to the definitive diagnosis of DEC. In two cases, the diagnosis of DEC was established by endometrial biopsy. Of the 68 cases, only one case was diagnosed with DEC after a bone metastasis was detected.

Lymphadenectomy was performed in only eight patients during the surgery; of these, four were of pelvic and four were of pelvic and para-aortic LNs. Adjuvant treatment was instituted in 25 cases; of these, 17 received only chemotherapy, four received only radiotherapy, and four received chemotherapy and radiation therapy. There was no consensus associated with the regimen of adjuvant chemotherapy for DEC, and the patients received one of the following regimens: cisplatin + anthracycline + taxane, taxane + carboplatin, cisplatin + anthracycline, or cisplatin + anthracycline + cyclophosphamide. For the two cases presented in our study, case 1 received systemic adjuvant chemotherapy with taxane + carboplatin and case 2 received best supportive care due to direct tumor invasion to the bladder and rectum and distant metastases.

Of the 34 cases with outcome data (including the two cases described in this study), 21 patients (62%) died as the result of the tumor, 7 (20%) were alive with the tumors, and 6 (18%) were disease-free. Case 1 was disease-free for 15 months after the initial surgery, and case 2, who had advanced disease, died a month after being admitted to the hospital. Zaibo et al. also reported the outcome data for 10 of 13 patients with DEC [9]. Of these, nine cases had recurrent or metastatic diseases within 3 years since their diagnosis. From this report, it is possible that DEC is associated with worse prognosis compared with the findings in our study.

Preoperative diagnosis of DEC is difficult by only endometrial curettage [8]. Our literature review showed that almost all cases were diagnosed by surgical specimens and only two cases were diagnosed by endometrial biopsy. In our study, endometrial curettage failed to establish the diagnosis of DEC; the pretreatment diagnosis for case 1 was adenocarcinoma and for case 2 was carcinoma. Considering the diagnosis criteria of DEC, it is difficult to establish the diagnosis by endometrial curettage sometimes. While the differentiated components of the tumor can be seen on the surface, the UC components are seen deeper in the myometrium; hence, the definitive diagnosis of DEC needs to be established from a specimen sample large enough.

For the diagnosis of DEC, it is important to differentiate UC component from high-grade endometrial cancer. The point is whether to have a foci of gland formation, and immunohistochemical studies are beneficial in conducting differential diagnosis. Differentiated components are strongly positive for keratins, epithelial membrane antigen (EMA), ER, and PR, whereas undifferentiated areas show almost complete loss of expression of these markers or only focal staining for keratins and EMA [6, 9].

The following entities should be considered in the differential diagnosis of the undifferentiated component of DEC: high-grade endometrial carcinoma, neuroendocrine carcinoma, unclassified sarcoma, and carcinosarcoma [13, 16]. The lack of focal expression for keratin or EMA in a tumor composed of oval cell of medium or large size arranged in sheets can be misinterpreted as an evidence of sarcoma. Most sarcomas, however, are composed of epithelioid and spindle cells. In addition, the expression of desmin, caldesmon, and smooth muscle actin is the key for differentiating sarcomas from the UC component of DEC because these markers are positive in sarcomas but negative in the UC component of DEC. Carcinosarcoma is composed of high-grade endometrioid carcinoma, with serous carcinoma being the most frequently observed histological type, which mainly comprises spindle-cell proliferation. In contrast, gland forming components are confirmed in DEC; these components indicate low-grade endometrioid carcinoma. The UC components of DEC can show neuroendocrine feature by expressing highly focal neuroendocrine-related markers. In contrast, neuroendocrine carcinoma shows strong and diffuse staining for neuroendocrine markers.

An association between DEC and Lynch syndrome has been previously reported [6]. Yokomizo et al. examined the immunohistochemistry for DNA mismatch-repair (MMR) proteins in three cases diagnosed as DEC and demonstrated the loss of MMR protein expression in the UC components of these patients [8]. Thus, the authors emphasized the importance of assessing the genetic background of patients with DEC. The two cases in our study had no family history of cancer.

DEC expresses aggressive clinical features, and the prognosis of these patients is poor [1, 17]. In our study, case 2 presented with the invasion to the bladder, rectum, and LN metastases. In contrast, the tumor in case 1 advanced into the endometrial cavity, similar to an endometrial polyp, without myometrial invasion. Hence, the diagnosis was established early. However, because DEC is typically associated with poor prognosis, we will continue to closely monitor this patient.

Because the criteria of DEC have been established recently, the incidence of DEC is unknown yet. Most studies have reported an incidence of UC to be approximately 1%–2% [18]. However, Altrabulsi et al. reported an incidence of 9%. They found that most cases of endometrium UC were mixed with endometrioid carcinoma; of the 56 cases of UC in the study, 40 (71%) were mixed with endometrioid carcinoma [5]. This evidence reinforces the need of a specimen sample to accurately establish the diagnosis of DEC. When surgery is not indicated due to advanced disease, the correct diagnosis of DEC may only be established through autopsy, as it occurred in case 2; it also can be diagnosis from the specimen of hysterectomy. Laura J et al. examined 32 carcinomas with UC components (26 endometrial and 6 of ovarian origin). Of the 26 endometrial cases, 10 (38.5%) showed the presence of an adjacent well-differentiated carcinoma [19]. DEC might be under recognized, and it is possible that the incidence of DEC is much higher than that previously reported.

## 4. Conclusion

Based on these studies, it is possible that there are cases of uterine endometrial cancer with DEC that are still undiagnosed due to advanced disease or aggressive tumor behavior, suggesting that the incidence of DEC is higher than that reported in previous studies. While we strive to improve the diagnosis of DEC, it is also crucial to better assess the prognosis and the appropriate treatment for the patients with established diagnosis of DEC.

## Figures and Tables

**Table 1:** Cases of dedifferentiated endometrial carcinoma (DEC).

Author	Year	No. cases	Age	Stage (cases)	Treatment (cases)	Adjuvant therapy (cases)	UC component, %	Outcome (cases)
J. Han [7]	2017	4	54	I	ATH+BSO+PLA+PALA	Not treated	30	NED (19 monhts)
			77	II	ATH+BSO+PLA	Not treated	20	DOD (7 weeks)
			52	II	ATH+BSO+PLA+PALA	Radiation (EBRT+ICR)	60	NED (39 months)
			60	III	SRH+BSO	Chemotherapy (CDDP+ADM+CPA) and Radiation (EBRT+ICR)	90	DOD (10 months)
R. Yokomizo [8]	2017	3	66	I	ATH+BSO+PLA+PALA ATH+BSO+OMX+	Chemotherapy (CDDP+ADM)	45	NED (24 months)
			48	III	Right hemicolectomy+ Hartmann operation	Chemotherapy (PTX+CBDCA)	90	DOD (5 months)
			48	IV	ATH+BSO+OMX+PLA+PALA	Radiation (EBRT)	60	DOD (7 months
Z. Li [9]	2016	13	61 (median)	I/II (1)	NA	Chemotherapy		NA
				III/IV (12)	NA		NA	DFI(>3 months) 10%
C. J. Stewart [10]	2015	17	68.5 (mean) (range: 49-86)	I (7)	NA	NA	NA	NA
				II (1)				
				III/IV (9)				
E. S. Wu [11]	2013	1	62	IV	T10-T12 laminectomy	Radiation and hormonal therapy (TAM)	NA	NA
R. Berretta [12]	2013	1	67	IV	ATH+BSO+Adrenalectomy	Chemotherapy (PTX+CBDCA)	NA	NA
Y. Shen [13]	2012	1	51	II	ATH+BSO+PLA	Vaginal radiation and chemotherapy (CDDP+DTX+Taxane)	20	NED (11 months)
G. Giordano [14]	2012	2	83	I	ATH+BSO+PLA	NA	almost all	AWD (12 months)
			61	III	ATH+BSO+PLA	NA	85	DOD (3 months)
G. Vita [15]	2011	1	45	III	ATH+BSO	Chemotherapy (CDDP+ anthracycline+taxane)	40	NA
E. G. Silva [1]	2006	25	51 (median) (range: 30-82)	I (14)	ATH+BSO (24)	Chemothrapy (18) Radiation (4) Not treated (3)	20-90	DOD (median 7 months)(15)
				II (1)				AWD (6)
				III (6)				NA (3)
				IV (4)				NED (104 months)(1)
